# A Case in Which a Urethral Catheter Could Be Indwelled by an Anterograde Approach After It Was Difficult to Indwell at the Start of Robot-Assisted Laparoscopic Radical Prostatectomy

**DOI:** 10.7759/cureus.62956

**Published:** 2024-06-23

**Authors:** Yasutomo Suzuki, Naoto Hodotsuka, Eigo Kuribayashi, Shuma Endo, Yukihiro Kondo

**Affiliations:** 1 Urology, Nippon Medical School Chiba Hokusoh Hospital, Chiba, JPN; 2 Urology, Nippon Medical School, Tokyo, JPN

**Keywords:** robot-assisted, radical prostatectomy, urethral stenosis, anterograde approach, pseudo-urethra

## Abstract

A case in which a urethral catheter could not be indwelled at the start of robot-assisted laparoscopic radical prostatectomy (RARP) is reported. A 64-year-old man was admitted to the hospital for RARP with a diagnosis of prostate cancer cT2aN0M0. At the start of RARP, a pseudo-urethra was formed by inserting a urethral catheter, so surgery was started with a transabdominal posterior approach without indwelling the urethral catheter. The urethra was opened during bladder neck resection, a guide wire was inserted anterogradely, the urethra was dilated retrogradely, and a urethral catheter was indwelled. After that, the procedure was performed as usual, and the operation was completed. When the urethral catheter could not be indwelled at the start of RARP, it was possible to do so using an anterograde approach during the operation.

## Introduction

During robot-assisted laparoscopic radical prostatectomy (RARP), urethral catheter indwelling at the start of surgery is essential for urine drainage and obtaining a prostate shape [1]. A case in which a urethral catheter was difficult to indwell at the start of RARP and a urethral catheter was later indwelled during the operation using an anterograde approach is presented.

## Case presentation

**Figure 1:**
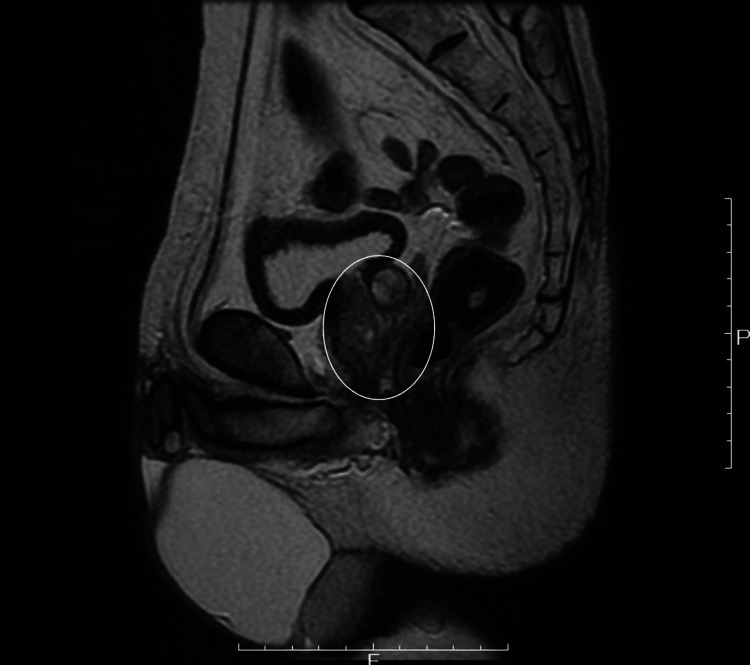
Sagittal MRI findings The white circle represents the prostate. No prostate enlargement was observed.

The case involved a 64-year-old man with no major complaint. A prostate biopsy was performed with a prostate-specific antigen level of 6.17 ng/mL, and the pathological result was adenocarcinoma, Gleason score 4+5=9. Following a close examination, he was admitted to the hospital for RARP with a diagnosis of prostate cancer cT2aN0M0. His history included pulmonary emphysema and depression. Preoperative magnetic resonance imaging showed a prostate volume of 21 ml (Figure 1). No obvious lower urinary tract symptoms were observed.

**Figure 2:**
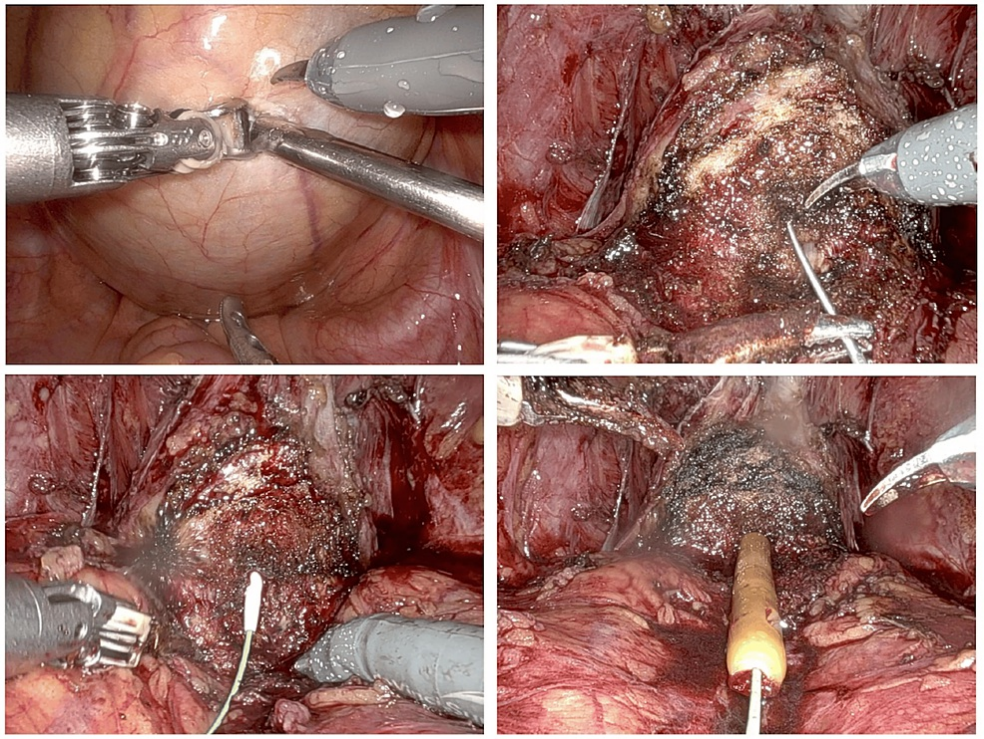
RARP findings Figure 2a: An incision is made in the posterior wall of the dilated bladder, and urine is aspirated. Figure 2b: After dissecting the ventral side of the bladder neck, a guide wire is anterogradely inserted from the incised urethra. Figure 2c: The urethra is retrogradely dilated with a pencil-type dilator using a guide wire. Figure 2d: The urethral catheter is indwelled retrogradely.

After the induction of general anesthesia, indwelling a urethral catheter was attempted; however, it was not possible to insert using a stylet because of resistance near the bulbous urethra by a non-Japanese board-certified urologist. An endoscope was used by a Japanese board-certified urologist. A false urethra was formed, and the guidewire could not pass through. Six ports were constructed transabdominally without a urethral catheter, and console operation was initiated. After an incision was made in the posterior wall of the bladder and the accumulated urine was aspirated (Figure 2a), the seminal vesicle and spermatic cord were detached by a posterior approach, and the retroperitoneal cavity was expanded. After separating the bladder neck and the ventral side of the prostate while confirming the shape of the prostate, the exposed urethra was opened, and the guide wire could be passed gracefully from the internal urethra to the external urethra (Figure 2b). A pencil-type dilator was used to retrogradely dilate the urethra from 8 Fr to 16 Fr using a guide wire (Figure 2c), and a 14Fr urethral catheter was indwelled (Figure 2d). There was slight resistance during urethral dilatation, and the presence of urethral stenosis was suspected. Subsequent procedures were performed as usual, and the prostate was resected. The incised posterior wall of the bladder was sutured with a 3-0 absorbent thread. Bladder-urethra anastomosis was performed without any particular problems, and the operation was completed. The operation time was 233 minutes, the console time was 197 minutes, and the amount of bleeding was about 10 ml. The patient’s postoperative course was uneventful. Five days after the operation, cystography was performed to confirm that there was no leak, the urethral catheter was removed, and the patient was discharged from the hospital. The length of hospital stay was 11 days. The pathological finding was pT2b, Gleason score 4+5=9, EPE0, RM0, adenocarcinoma of the prostate. One year after the operation, no complaint of lower urinary tract dysfunction would suggest urethral stenosis. 

## Discussion

In the present case, iatrogenic urethral injury prevented the indwelling of a urethral catheter. Iatrogenic urethral injury, as in the present case, is a complication that occurs at a relatively high rate of 0.67% [2]. If a urinary catheter cannot be indwelled using standard techniques, a stylet is used as the next step. However, it is important to perform retrograde urethral catheter indwelling using urethroscopy without overdoing it because a pseudourethra is formed due to blind technique, and it becomes difficult to then indwell a urethral catheter [3]. However, a urethral catheter indwelling using a stylet carries the risk of pseudourethra formation due to the blind technique, which makes subsequent urethral catheter indwelling using a urethroscope difficult [3].

When the urethral catheter cannot be indwelled retrogradely, it is common to construct a cystostomy [4], but in the present case, it was not possible because some ports were constructed after that.

In RARP, which is a posterior approach without a urethral catheter, the dilated bladder obstructs the field of vision. Therefore, this time, the posterior approach was possible by incising the posterior wall of the bladder and then aspirating the urine. The problem with the transection of the bladder neck is that the shape of the prostate cannot be grasped by pulling the urethral catheter. In the present case, at the stage of bladder neck treatment, it was possible to recognize the bladder neck relatively easily by using the method of Shimbo et al. [5]. After opening the internal urethra, a guide wire was passed through the urethra antegradely; the urethra was then dilated, and the urethral catheter was indwelled retrogradely. This method is often reported as the primary endoscopic realignment of urethral injury associated with pelvic fractures [6-9], suggesting that it was a very useful procedure in cases such as the present one.

Although no lower urinary tract symptoms were observed in this case after surgery, urethral injury may be a risk factor for urethral stenosis. Therefore, retrograde urethrography and careful follow-up of changes in lower voiding symptoms are necessary in the future.

## Conclusions

Generally, it is essential to indwell the urethral catheter at the start of RARP, but we have encountered a case in which a urethral catheter could not be indwelled at the start of surgery. In such cases, a catheter can be indwelled intraoperatively using an antegrade approach through the open urethra after bladder neck transection. This is the first report of this method, and we believe it will be very useful in similar cases in the future.
